# Biology wet lab e‐learning during and after the COVID‐19 pandemic: A review of student learning and experiences

**DOI:** 10.1002/bmb.21897

**Published:** 2025-03-27

**Authors:** Cristina Maglio, Manuela Williams, Alessandro Camponeschi

**Affiliations:** ^1^ Department of Rheumatology and Inflammation Research Institute of Medicine, The Sahlgrenska Academy at University of Gothenburg Gothenburg Sweden; ^2^ Department of Rheumatology Sahlgrenska University Hospital Gothenburg Sweden; ^3^ Department of Humanities University of Strathclyde Glasgow UK; ^4^ Department of Clinical Immunology and Transfusion Medicine Sahlgrenska University Hospital Gothenburg Sweden

**Keywords:** e‐learning, biological science, learning, student experience, student perceptions, teaching, wet lab

## Abstract

The COVID‐19 pandemic began as a health crisis and quickly turned into an economic, social, and political crisis. It revealed the vulnerability of education systems to external changes and risks and challenged institutions and educators to transform and adapt at short notice. Following the COVID‐19 outbreak, one of the natural consequences was the unprecedented rise in online education. The transition from the in‐person teaching format to e‐learning exposed teachers and students to significant challenges. In the biomedical field, e‐learning forced teachers to rethink hands‐on wet lab teaching into a hands‐off virtual one; this digital transformation has continued in the post‐pandemic period and has resulted in the emergence of hybrid models trying to harmonize the benefits of e‐learning with those of in‐person teaching. In this narrative review, we analyzed articles published between 2020 and 2024 focusing on the teaching of molecular and cellular biology laboratory through online or blended learning formats. We focused on the impact that pedagogical innovation in laboratory e‐learning has had on student perceptions, experience, and outcomes. We have extracted five major themes that should be considered by educators involved in course design to enhance the benefits of exposing students to learning in a virtual lab: (1) the varying effectiveness of laboratory e‐learning, (2) the potential for online labs to foster self‐efficacy and confidence, (3) the reduced opportunities for social interaction in virtual settings, (4) students' perspectives on virtual, blended, and in‐person lab work, and (5) the importance of addressing student inequities in digital access.

## 1 INTRODUCTION

On March 11, 2020, a global pandemic of the Coronavirus Disease 2019 (COVID‐19) was declared by the World Health Organization (WHO). Many higher‐education institutions (HEIs) were forced to make rapid changes to the delivery of the curriculum, moving their courses to online platforms and exploring new ways of teaching and learning. Such a transition to remote learning environments, although accelerated by the COVID‐19 pandemic, was part of a trend that had already begun in some institutions prior to 2020.^1^, ^2^, ^3^ Course management systems, such as Canvas or Google Classroom, which could be adapted to online learning, helped facilitate the transition from in‐person to online teaching during the pandemic.

In the year prior to the pandemic, educators had mixed opinions over the use of e‐learning, and an echo of those concerns informed academic debates during and after the pandemic; some educators believed that the lack of social interactions and potentially impaired communication skills were among the most significant and long‐term disadvantages of e‐learning.^4^ Others were confident that blended teaching/learning combining in‐person with e‐learning was the most efficient way to proceed, as it would give the students flexible ways to access course material and at the same time expose them to information from several angles.^5^, ^6^, ^7^, ^8^ Furthermore, the flexibility and accessibility offered by e‐learning appeared to have benefited a diverse student population and enabled students to structure their learning at their own pace and in an environment where they felt comfortable.^9^ The COVID‐19 pandemic forced individuals and institutions to re‐evaluate their approaches and experiences and find new ways of designing learning activities that promote student participation.^10^

Hands‐on laboratories are an integral part of the science curriculum and play a central role in the learning process across all areas of study, engaging students in educational activities and fostering problem‐solving and critical‐thinking skills. Moreover, these labs provide the students with a deeper understanding of scientific concepts by allowing them to apply theoretical knowledge in a practical setting. A wet lab, or experimental lab, is a laboratory equipped with all the tools to allow a wide range of hands‐on scientific research and experiments. Usually, wet labs allow safe handling of different types of potentially dangerous chemicals. This is certainly the case of molecular and cellular biology laboratories, which are designed to study biological processes governing the physiology of the cell and orchestrated by molecules. The investigation of those cellular mechanisms requires the knowledge of several, often challenging, techniques that are learned during laboratory classes. Students attending these laboratories learn some of the basic techniques ranging from pipetting to titration and cell culture, data acquisition with several instruments such as microscope, flow cytometer, and thermocycler, and, eventually, data analysis and interpretation. Gaining proficiency in these skills enhances confidence, improves data accuracy, and prepares students who wish to pursue a career in science.^11^

Traditionally, hands‐on lab experiences have been believed to foster students' interests towards biology learning whilst offering the opportunity to deveolop a wider set of skills and competencies in an interactive environment.^11^, ^12^ The learner's experience in a traditional science wet lab can be defined by four types of interactions: 1. student‐to‐student interaction, which happens within or between groups of students; 2. student‐instructor interaction; 3. student‐equipment interaction, when students manipulate equipment; 4. vicarious interaction, when students learn by observing others. The first two are interpersonal and result in two‐way communication, which often includes instant response/feedback.^13^ Research has outlined that students value face‐to‐face feedback and gain motivation from their direct interaction with peers and instructors; group interaction also fosters metacognition, the understanding of one's process of thinking and learning, and the handling of lab equipment contributes to developing a sense of responsibility.^11^, ^12^

Research into the use of wet lab e‐learning to help students acquire and develop practical skills in the field of biology predates the spring of 2020. Studies show that students' perceptions of the experience and usefulness of the virtual environment varied; whilst many mostly saw e‐learning as a complementary alternative to the more traditional activities taught in person,^14^ others had a clear preference for hands‐on lab experience.^15^ For example, a few months before the beginning of the pandemic, a study from the Kansas City University of Medicine and Biosciences investigated the perception of medical students of online vs. in‐person microbiology wet lab learning.^16^ Students were divided into two groups and an online survey was administered at the end of the study; this revealed that, although students broadly appreciated the benefits of lab e‐learning, they also preferred some form of in‐person interaction or a blend of online and in‐person activities.

### 1.1 E‐learning approaches: Self‐paced and instructor‐led

Over the past decade, a number of e‐learning approaches have been developed and used alongside in‐person teaching, which have built on students' existing digital skills. With the growing diversity among students, virtual learning environments and hybrid teaching models have offered opportunities for more flexible delivery.^7^ The deployment of these new e‐learning approaches was dramatically accelerated during the period 2020–2021 by the COVID‐19 pandemic and the closure of university campuses.

E‐learning approaches include the self‐paced and the instructor‐led methods.^17^ In self‐paced e‐learning, pre‐made material such as videos or presentations is provided to the students before the course. This method is also defined as “asynchronous” because the material can be used whenever the student wants, and not necessarily within scheduled teaching activities.^18^ This setting has the advantage of being flexible, also giving the possibility to repeat concepts that were not clear and to follow lectures at an individual pace. This approach may be more affordable than the instructor‐led one, as it can reduce the need for teachers to be present during the learning process, though this is not universally the case across all institutions. However, the lack of teacher‐student interaction does not give the opportunity to the student to ask questions straight away, and this can limit the learning experience as well as opportunities to build communities of learners. In the instructor‐led approach, the course is scheduled and led by a teacher through an online platform; this is often defined as “synchronous” e‐learning process.^19^ Here the events take place in real time and require both teacher and student to be present online at a given time. This setting may have a higher cost than the self‐paced one, but the presence of the teacher during the learning process has also clear advantages as the student can ask questions in real time and the teacher can provide answers and support to students in developing solutions.

## 2 MATERIALS AND METHODS

**FIGURE 1:**
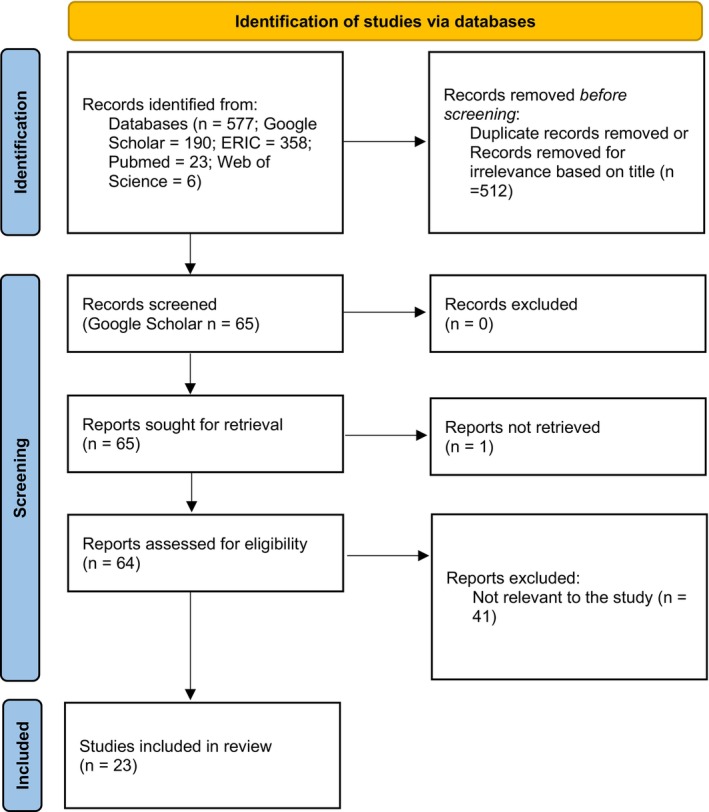
PRISMA flow diagram of the systematic review process. A total of 577 records were identified across four databases. After removing 512 duplicates and irrelevant titles, 65 records were screened, and 64 were assessed for eligibility. Following exclusions, 23 studies were included in the review, focusing on wet lab e‐learning and students' experiences during the COVID‐19 pandemic.

A systematic literature search was conducted to identify relevant original articles on the topic of wet lab and e‐learning during and after the COVID‐19 pandemic that presented the students' point of view and feedback. The search was performed across multiple electronic databases: Education Resources Information Center (ERIC), Google Scholar, Web of Science, and PubMed. Citation chaining through backwards searching from the retrieved literature was used to identify material for further consideration. Search terms included combinations of keywords such as “wet lab,” “laboratory,” “bench lab,” “e‐learning,” “biology,” “COVID‐19,” “teaching,” “students' experience,” “students' outcomes,” and “students' achievements.” The search was restricted to English‐language original research articles published in peer‐reviewed journals between March 2020 and October 2024. The retrieved articles were screened based on their relevance to the topic. Only articles that specifically addressed the challenges, strategies, and outcomes of teaching molecular and cellular biology laboratory techniques in an online or blended learning format were included. Studies were excluded if they focused only on surgical, dentistry, or pathology procedures, were not conducted in HEI, lacked data on students' experiences, or were reviews and meta‐analyses. After removing duplicates and articles irrelevant based on title or exclusion criteria, we screened 577 original articles. Of these, 23 met the inclusion criteria and were included in the review (Figure 1).

### 2.1 Ethical considerations

This article is a review of existing literature on the students' experience of online and blended wet‐labs. The research did not involve human participants, and ethics approval was not required.

## 3 REVIEW

Since 2020, several short reports, studies, and trials investigating the impact of wet lab e‐learning experience during and after the COVID‐19 pandemic have been published. Some articles, published slightly after the beginning of the pandemic, addressed the sudden transitioning process to online‐only wet‐lab programs that many HEIs experienced and reported the strategies that were implemented to face the challenges.^20^, ^21^, ^22^, ^23^, ^24^ Many studies gave suggestions on how to move specific wet lab teaching to online‐only platforms.^22^, ^23^, ^25^, ^26^ A non‐exhaustive list of commonly used online platforms and resources for virtual labs is shown in Table 1. In our narrative review, we focus on a selection of reports that, by virtue of their originality or comprehensive analysis of students' perceptions are more representative of the challenges faced during the first phases of the COVID‐19 pandemics in terms of the pedagogical framework designed to support the delivery of wet lab teaching and of the impact that the emergence of online teaching and subsequent hybrid models have had on student learning and experience. Five key themes emerged from the review; each theme addresses both students' perceptions and student learning outcomes, highlighting how online or blended lab instruction can be most effectively designed.

**TABLE 1:** Commonly used online platforms and resources for virtual labs.

Online platforms and resources	Website
Beyond Labz	https://www.beyondlabz.com/
BioInteractive	https://www.biointeractive.org/
ChemCollective	https://chemcollective.org/vlab/104
Edvotek	https://www.edvotek.com/Experiments/mylab‐distance‐learning?srsltid=AfmBOop6cRa5gHyalrTdCncNoUQzUbT‐maR_hF8_‐UpdRmK2_XLEDYq0
Journal of Visualized Experiments (JoVE)	https://www.jove.com/
LabBuddy	https://www.labbuddy.net/
Labster	https://www.labster.com/
LabXchange	https://www.labxchange.org/library
PhET Interactive Simulations	https://phet.colorado.edu/
PraxiLabs	https://praxilabs.com/
Virtual Biology Lab	https://virtualbiologylab.org/
VirtualLabs	https://www.vlab.co.in/participating‐institute‐amrita‐vishwa‐vidyapeetham

### 3.1 The varying effectiveness of laboratory e‐learning

Educators were investigating the effect of virtual labs on student learning before the outbreak of the COVID‐19 pandemic in 2020. In 2018, a study comparing hybrid wet/virtual lab curriculum vs. a traditional curriculum for general chemistry was also conducted at a master's granting university in the Pacific Northwest (the article does not state the name of the university but the authors are affiliated with Western Washington University), showing that replacing part of traditional wet laboratories with virtual ones does not compromise students' learning.^1^ Other studies have shown that in a blended learning setting, students perform marginally better than in a face‐to‐face environment and that online learning appears to be as effective as in‐person learning; when differences are present, they are often due to wider personal and environmental factors that affect the learning process, rather than the mode of delivery.^5^, ^6^

An investigation into the outcome and experience of students at an American introductory college biology CURE in 2021 compared three main instructional modalities: face‐to‐face, hybrid and online. The results appeared to support a widespread perception that face‐to‐face was the most effective learning modality and online the least effective: when considering student outcomes, online students appeared disadvantaged compared to face‐to‐face students and hybrid students, while hybrid students did not appear to learn to analyze the data as competently and confidently as face‐to‐face students.^27^ Conversely, several other studies have extolled the benefits of virtual lab learning. Serrano‐Perez et al., for example, outline how online lab teaching encourages teamwork, facilitates active learning and fosters creativity,^28^ while Quin et al.'s study reveals that the use of virtual reality in the teaching of online labs made learning interest‐provoking, “more tangible and playful”.^29^ The informative and enjoyable aspects of learning in online labs were also noted by Lau et al.^30^

A controlled observational study from 2020 reported the effect of virtual labs on students' achievement at Medical and Dental Faculties at King Salman International University, South Sinai, Egypt. The control group, that received lab training in a conventional way, achieved significantly better scores in a qualitative analysis of carbohydrates compared to the intervention groups (92% for traditional lab training vs. 70% for pre‐lab training followed by virtual labs vs. 53% for pre‐lab only, *p* < 0.001). However, for protein detection assessment, students using the virtual lab scored significantly higher on average (91%) than those trained in real labs (85%) or through instructional videos (62%, *p* < 0.001). The authors also report that the students' achievement was overall elevated. In general, the students who attended virtual labs appreciated the flexibility and the clarity of e‐learning, although they mostly preferred a traditional lab practice.^31^

The differences observed in these studies highlight that virtual labs may be particularly effective for tasks requiring repeated experimentation, such as protein detection, where students can practice independently and refine their understanding. However, tasks that require complex, hands‐on manipulation, such as qualitative carbohydrate analysis, may still benefit from the tactile experience of traditional labs. Nevertheless, these findings also highlight that the effectiveness of virtual labs may depend on the nature of the lab techniques being taught and the specific learning outcomes. Taken together, these studies illustrate that while virtual labs can sometimes meet or exceed the effectiveness of traditional labs for particular assessments or under specific conditions, they do not universally replicate the full depth of hands‐on experiences. This underscores the importance of matching instructional design to the learning goals of each lab activity.

### 3.2 The potential for online labs to foster self‐efficacy

Self‐efficacy, the individual's belief in their capabilities to achieve specific goals, is an important aspect of the student learning that appears to be facilitated by virtual labs. Self‐efficacy is also regarded as a predictor of performance.^27^ Virtual and hybrid laboratories and experiments have been shown to promote self‐efficacy, engagement and self‐regulation, which lead to greater student engagement and satisfaction.^27^, ^32^ In an in‐person wet lab, experiments are usually conducted once in a limited timeframe, whereas in a virtual lab, experiments can be repeated multiple times until complex concepts become clear, helping students develop confidence in their techniques.^33^, ^34^ Keles et al.'s study concludes that virtual labs are effective tools to prepare students for real labs; interestingly, two‐thirds of the students surveyed by the authors claim that simulation labs are particularly beneficial when used immediately before a theoretical lecture.^33^ This repeated practice in a low‐stakes environment can further bolster students' self‐efficacy.

Another example of virtual labs enhancing self‐efficacy is presented by Choudeva and Soliman who explore staff and student's perceptions of Beyond Labz, a digital simulation platform built upon real experimental data, and which offers chemistry, organic chemistry, biology, physics and physical science labs. The study was developed at an Ontario polytechnic college in the winter of 2021; it argues that Beyond Labz is a useful tool to supplement in‐person labs and it prepares students for real‐life experiments after exploring options and possibilities online. Staff interviewed in the study comment that “Students can learn to run an experiment, make mistakes, and practice developing critical thinking and problem‐solving skills before getting to the “wet” lab.^35^ This helps them become active learners and develop deeper critical thinking and reflective skills.

Moelans et al. demonstrated that the success of laboratory e‐learning environments, such as LabBuddy®, is closely linked to students' self‐regulated learning abilities.^32^ These skills enable students to effectively manage their time, set goals, and monitor progress, which not only increases their engagement and satisfaction with the virtual experimental environment but also positively impacts their confidence and learning outcomes. Clear instructions and structured guidance within e‐learning environments further empower students to confidently approach both virtual and in‐person tasks. As these examples suggest, virtual labs can create a space for students to practice repeatedly, make errors safely, and gradually build the skills and confidence they need for real‐life experimentation.

### 3.3 Reduced opportunities for social interaction

The process of learning not only involves the acquisition of information but also the development of a wider set of skills, competences, and behaviors. A significant challenge posed by e‐learning, particularly during the pandemic, was the reduced opportunities for social interaction. Howard Wolinsky outlines the lack of physical interaction in an online learning environment as a considerable challenge for learners.^36^ Attending classes together enables students to form groups of friendship and support that exist outside the classroom; these social groups allow students to carry on discussing what happened in class and, through this dialogic process, students continue to learn. The article emphasizes that the learner experience at university is “transformative” and not limited to mastering content and passing tests. The teaching of natural sciences in laboratories allows students to work in pairs or small groups and learn about cooperation and collaboration in a manner that cannot be easily replicated online; educators interviewed in this article noted that online platforms like Zoom significantly reduced communication within the class, as students tended to switch their cameras off and mainly communicate with the lecturer through the chat tool.

It is evident when looking at other studies that virtual lab experience cannot entirely replace the learner's experience in a real lab, primarily because of the lack of one‐to‐one interaction between student and educator; instead, there seems to be emerging consensus around the usefulness of virtual labs as a preparation for hands‐on physical labs.^33^, ^37^ This consensus has also built on pre‐pandemic studies that have shown that the use of virtual labs is as efficient as face‐to‐face tutorial in preparing the students for a following “in real life” physical lab experience, emphasizing that a combination of virtual and “hands‐on” is the way forward for science education.^27^, ^36^

Similarly, a survey conducted at the Dept. of Molecular Biology and Genetics of the Democritus University of Thrace, Greece, during spring 2020 reported that the students considered e‐learning exciting and flexible, but they missed social interaction with fellows and teachers.^38^ Some studies have further outlined students' overall preference for a higher number of traditional lab experiments, where increased student motivation was recorded, and a lower number of virtual experiments, although it is apparent that any type of lab instruction and interaction is perceived as very valuable by the students.^28^

### 3.4 Students' perspectives on virtual, blended, and in‐person lab work

Students' experience of learning in wet lab was affected by the restrictions introduced during the COVID‐19 pandemic. Whilst the in‐person lab remained the preferred options, up to 91% of chemistry students at the University of Leicester appreciated the extra support received to prepare for the experiments. At Leicester, educators noted that blended learning models were more conducive to higher student satisfaction and that anonymised student contribution resulted in greater engagement and participation; finally, they recommended robust training to help students familiarize with the relevant technologies.^39^ Clear pre‐recorded instructions have also been reported as having a positive impact on how students experienced learning and performed in a hybrid laboratory at Southern Arkansas University, where the blended format remained the students' preferred modality.^40^

In 2020, Hsu and Rowland‐Goldsmith reported a survey of student perceptions of the online teaching transition between spring 2019 and spring 2020.^41^ Students of a first‐year molecular genetics course at a private institution in southern California were surveyed after the last day of classes in both spring 2019 and spring 2020. The course included a lecture and a lab component. The students did not report significant changes in the factors that contributed to their learning in the lab component of the course, but in the 2020 survey they cited the inability to complete the hands‐on aspect of the lab as the most common barrier to their learning, followed by the online learning itself. In 2020, there was also a significant increase in students reporting that it was harder to engage online, whether in breakout groups or with the class.^41^

### 3.5 Addressing students' inequities in digital access

Course design and access to technology and resources are key to students' participation in online lab and improved outcomes. Delgado et al. argue that whilst it is not feasible for instructors to reproduce the in‐person lab experience online, there are “creative solutions” that can be introduced when designing a course and which can offer “valuable scientific learning experiences” to students.^21^ Dustman et al. outline that, despite the benefits lab simulation offers to student engagement and learning, online learning can also present some challenges to students; for example, inequalities in accessing digital technologies mean that some students may not be able to access and benefit from learning in virtual lab modules.^42^, ^43^ Chudaeva and Soliman's work emphasize the impact of the digital divide on students' access to hardware and internet connection even when the software was provided; where students could not access the necessary resources, they were given alternative individual or group assignments.^35^ The study also outlines the importance of scaffolding virtual learning to support learners who may have not had experience of online platforms.

A thorough integration strategy and a robust pedagogical approach are needed to ensure the success of online teaching activities, particularly those based on virtual reality tools, which often strain the scaffolding of learning in a syllabus that is primarily based on traditional activities such as lectures, in‐person laboratories, and group work.^29^ However, careful positioning of online lab activities and designing of the teaching material can greatly enhance the experience of students and their interaction with the course. Scaffolding online learning with interactive material (such as computer simulation exercises and quizzes with immediate feedback) has shown to be an effective way of preparing students for the practical tasks, consolidating their understanding of the theory behind the lab activities, and developing subject‐specific competencies; the use of interactive material and activities as preparation for practical lab work also meant that students could work at their own pace, were able to internalize and act on initial feedback, and this resulted in fewer instances of reassessment.^44^

### 3.6 Limitations

For the purpose of this review, only material in English has been selected and included; indeed, within the literature there is some indication that the dominance of the English language in the software instructions for virtual lab activities may be placing non‐English speaking students at a disadvantage. Given the nature of the review, we did not produce a detailed analysis of students' responses and outcomes by gender, ethnicity, age, or socio‐economic grouping. Finally, more research is needed to assess the virtual learning experience of students with disabilities.

## 4 CONCLUSIONS

The COVID‐19 pandemic caught many institutions unprepared; the crisis highlighted the strengths and weaknesses of online learning and at the same time accelerated its growth dramatically, a process that had already gathered momentum during the last decade.^1^, ^2^, ^3^ Traditionally, laboratories have always been considered a synonym for hands‐on experience for the students, a taste of “real‐life science”; the sudden move to remote learning at the start of the pandemic highlighted to many students the benefits of the traditional face‐to‐face teaching, for example the fact that teachers can provide real‐time and immediate feedback to students because they are present in the same place at the same time. In short, while digital platforms introduce an element of convenience and adaptability, they cannot fully capture the immersive experience of hands‐on lab work. However, the move to online delivery of teaching that was seen as a temporary measure also spurred the development of innovative approaches to the design and delivery of wet labs remotely, which have been used in the post‐pandemic period to enhance the experience and learning of students of science degrees. All studies considered here have supported the adoption of a hybrid model, and not the wholesale replacement of in‐person teaching with remote delivery. A summary of the strengths and weaknesses of the different lab models analyzed in this review is presented in Table 2.

**TABLE 2:** Summary of the strengths and weaknesses of the different lab models.

	Traditional lab	Hybrid lab	Virtual lab
Strengths	Hands‐on skill development	Hands‐on skill development	Possibility to attempt experiments multiple times at own pace
	Interpersonal interactions	Interpersonal interactions	Availability anytime and anywhere
	Real time and immediate feedback	Possibility to attempt experiments multiple times at own pace	Flexibility
		Flexibility	Multifaced information
		Multifaced information	
		Real time and immediate feedback	
Weaknesses	High costs	High costs	No hands‐on skill development
	Time and resource consuming	Time and resource consuming	Limited trouble shooting
	Low flexibility	Affected by limited access to technology	Affected by limited access to technology

This review has noted the challenges educators faced as they attempted to recreate online the experience of hands‐on training, including the collaborative engagement between instructors and students and between students and their peers. However, what also emerges from the literature is a growing confidence in the benefits of virtual lab learning, which provides students with the opportunity to repeat experiments, make mistakes, and become confident in the techniques they have learned before they engage with in‐person lab work. Within this environment, the hybrid model of delivering lab experience has gained traction and has increasingly been regarded as the most appropriate to consolidate student learning.

This review has also highlighted some key issues that should be at the forefront of educators' approaches to the design and delivery of virtual lab teaching. The digital divide and inequalities in accessing technology and internet can have a detrimental impact on the way in which students experience remote learning and on the outcomes of their learning. Inequalities were particularly evident during the pandemic when students from underrepresented groups and from marginalized socio‐economic communities struggled to keep up with online learning not only because of the limited access to technology but also because of a lack of private space where to study at home and often because of competing family responsibilities.^43^ As many universities have seen their student population become increasingly diverse over the years, uneven access to resources must be considered by educators at the point when a new course and new activities are designed.

In order to support students who take part in virtual lab activities for the first time, scaffolding knowledge and experience of the relevant technology is very important, as is the positioning of online laboratory activities within the syllabus and the sequencing of traditional teaching activities such as lectures, seminars, and group work.

## FUNDING INFORMATION

This work was supported by the King Gustav V's 80‐year Foundation (SGI‐2018‐0510, FAI‐2019‐0618, FAI‐2020‐0706), Assar Gabrielsson's Foundation (FB21‐104), Adlerbertska stiftelsen, Amlövs Stiftelser, Göteborgsregionens Stiftelse för Reumatologisk Forskning to Alessandro Camponeschi, and by Svenska Sällskapet för Medicinsk Forskning (S20‐0109) to Cristina Maglio.

## CONFLICT OF INTEREST STATEMENT

The authors declare no conflict of interest.

## Data Availability

Data sharing not applicable to this article as no datasets were generated or analysed during the current study.
